# Hyperbaric Oxygen as an Adjunct in the Treatment of Venous Ulcers: A Systematic Review

**DOI:** 10.1177/15385744231162924

**Published:** 2023-03-08

**Authors:** Colum Keohane, Daniel Westby, Fiona C. Nolan, Mark Twyford, Wael Tawfick, Stewart R. Walsh

**Affiliations:** 1 Specialist Registrar, Vascular Surgery, 8799 National University of Ireland Galway, Galway, Ireland; 2 Clinical Lecturer, 8799 National University of Ireland Galway, Galway, Ireland; 3 Professor of Vascular Surgery University Hospital Galway, and Associate Director Lambe Institute for Translational Research, 8799 National University of Ireland Galway, Galway, Ireland

**Keywords:** hyperbaric oxygen, venous Ulcer, Leg Ulcer

## Abstract

**Background:**

The use of Hyperbaric Oxygen Therapy (HBOT) in diabetic wounds has been studied extensively. Even though venous insufficiency is the most common cause of lower limb ulceration, there is comparatively little evidence regarding the use of HBOT for Venous Leg Ulcers (VLU). We performed a systematic-review to evaluate and synthesise available evidence, to evaluate whether patients with VLU, when treated with HBOT, had greater rates of (i) complete VLU healing or (ii) reduction in VLU area, than controls.

**Methods:**

In keeping with PRISMA guidelines, database searches of PubMed, Scopus and Embase was performed. After removal of duplicates, titles were screened for relevance by two authors, then abstracts, and in turn full text manuscripts. Data were extracted from relevant sources including one published abstract. Included studies were assessed for risk of bias using the Risk of Bias 2 (RoB-2) and Risk Of Bias In Nonrandomized Studies (ROBINS-I) tools.

**Results:**

Six studies were included. There was significant heterogeneity across the studies, with no standard control intervention, method of outcome reporting, or duration of follow up. Two studies reported 12 week follow up results and pooled analysis of complete ulcer healing showed no statistically significant difference between HBOT and controls for the outcome of complete ulcer healing OR 1.54 (95%CI = .50-4.75) P = .4478. A similar non-signifiacnt result was seen in four studies reporting 5-6 week follow up; OR 5.39 (95%CI = .57-259.57) P = .1136. Change in VLU area was reported in all studies, and pooled standardised mean difference was 1.70 (95%CI = .60 to 2.79) P = .0024, indicating a statistically significant benefit of HBOT in reducing ulcer area.

**Conclusion:**

Existing evidence suggests that HBOT does not significantly affect complete healing of VLU. There is a statistically significant benefit in terms of reducing ulcer size, though in the absence of ulcer healing the clinical significance of this is not established. Current evidence does not justify widespread use of HBOT for VLU.

## Background

The use of Hyperbaric Oxygen Therapy (HBOT) as an adjunct in wound care is not a novel concept but the majority of research into this field focuses on ischaemic and in particular, diabetic wounds. The most common cause of lower limb ulcers however is Chronic Venous Insufficiency (CVI), and Venous Leg Ulcers (VLU) pose a massive burden on health services internationally. Literature surrounding the use of HBOT in VLU remains scant.

In ischaemic ulcers, or diabetic wounds where ischaemia is commonly a factor, it is easy to rationalise the benefit of HBOT. In VLU however the benefits are less intuitive. There is a sound scientific rationale however to suggest that hyperbaric oxygen may benefit patients with VLU. Reduced partial oxygen tension has been shown in the tissues surrounding VLU^1^ and while the precise mechanisms by which venous insufficiency leads to ulceration have not been fully elucidated, many of the prevailing explanations implicate local hypoxia.^2^

Chronic wounds of differing aetiology have many similar characteristics and heal by numerous similar processes and HBOT has been shown to have potential benefits in many of these. Acute inflammation requires the formation of a provisional fibrin and fibronectin extracellular matrix but this process must be halted appropriately, before normal tissue is damaged by the inflammatory response. This can happen when chronic inflammation disrupts normal repair, leading to excessive fibrosis. This inflammatory response can be down-regulated in response to Reactive Oxygen Species (ROS) and Reactive Nitrogen Species (RNS), which decrease synthesis of chemokines by monocytes and alter inflammatory modulators such as hypoxia inducible factor-1, Haem oxygenase-1, and heat shock proteins. ROS and RNS can also up-regulate neovascularisation by a combination of increased synthesis of growth factors within the wound, e.g., Vascular Endothelial Growth Factor^3^ and by increased mobilization from bone marrow of stem and progenitor cells.^4^ HBOT leads to increased intracellular oxygen, which produces an increase ROS and RNS. The effect of HBOT on ROS and RNS is independent of hypoxia, and so the effects of HBOT on chronic wounds should not be expected to be confined to hypoxic wounds. Excessive ROS production can lead to oxidative stress however, which has detrimental effects on wound healing and elevated, sustained ROS have been detected in vivo and have been associated with impaired wound repair in chronic, non‐healing wounds.^5^

HBOT is delivered across multiple sessions, the number of which can be quite variable. Likewise, the duration of sessions can vary from less than an hour, to four hours or more. Pressure within the chamber pressure is maintained between 2.5 and 3.0 atm for most uses. Acute therapy for decompression sickness of carbon monoxide poisoning usually only require a short course of longer treatments, while chronic wounds may require up to 40 or more sessions of shorter duration.^6^ There remains considerable heterogeneity in the process of delivering HBOT.

### Objectives

The objective of this review was to investigate if; in patients with VLU, the addition of hyperbaric oxygen to patients’ existing wound care was associated with improved healing vs continuing existing pre-study wound care alone.

## Methods

This systematic review was conducted in accordance with the Preferred Reporting Items for Systematic Review and Meta-Analysis (PRISMA) 2020 guidelines.^7^ Studies were considered eligible for inclusion if they reported original research comparing patients with an active VLU receiving HBOT, against a control group receiving the same therapy with the single exception of the HBOT. Studies were excluded if they were not published in English and no English language translation could be obtained, or if no abstract could be obtained.

There were two primary outcomes for this review; the proportion of VLU healed in the HBOT vs Non-HBOT groups, and reduction in VLU area in the HBOT vs Non-HBOT groups

### Search Strategy

The database search was carried out using three databases: PubMed, Scopus and Embase. No significant or landmark articles pertaining to the subject were known in advance so it was considered particularly important that smaller studies not be overlooked, as might happen with a more refined search strategy. Search terms were therefore intentionally kept broad. Searches sought any combination of the terms ‘Hyperbaric Oxygen’, ‘HBOT’ or ‘Hyperbaric O_2_’, with any of the terms ‘Vein’ or ‘Venous’ or ‘Varicose’ and the term ‘Ulcer’.

After removal of duplicates, titles were screened by two authors (CK and MT), blind to each other, and disputes mediated by a third author (FN). When a list of likely relevant titles was agreed, the same authors screened the abstracts of these. The same reviewers then independently extracted relevant data from the included studies.

Where results were presented at multiple time-points in an individual study, all data were extracted provisionally to allow synthesis of results at similar time-points where possible.

#### Risk of bias

The Risk of Bias-2 (RoB-2)^8^ tool was used to assess bias in the randomly allocated studies, while non-random studies were assessed using the Risk Of Bias In Non-randomized Studies (ROBINS-I).^9^ These were graphically represented using the Cochrane traffic light system.^10^

#### Synthesis

As a dichotomous variable, healing was reported as the Odds Ratio (OR) with 95% confidence interval (95%CI), and underwent meta-analysis using the OR. The change in VLU size, a continuous measurement, required an effect size meta-analysis. To achieve this despite some studies reporting the change in VLU size as absolute area, while others reported only percentage change, the Standardized Mean Difference (SMD) was used. Random effects models were used for both meta-analyses. Forest plots were used to graphically represent the synthesised data.

Due to the reporting of variable follow up periods two separate groups were devised based on follow-up duration. Where results were presented at different time-points within a study, the results which conformed most closely to one of these groups were used.

All statistical analysis was performed using StatsDirect Statistical Package version 3 (StatsDirect Ltd., Cambridge, UK).

## Results

**Figure 1.**
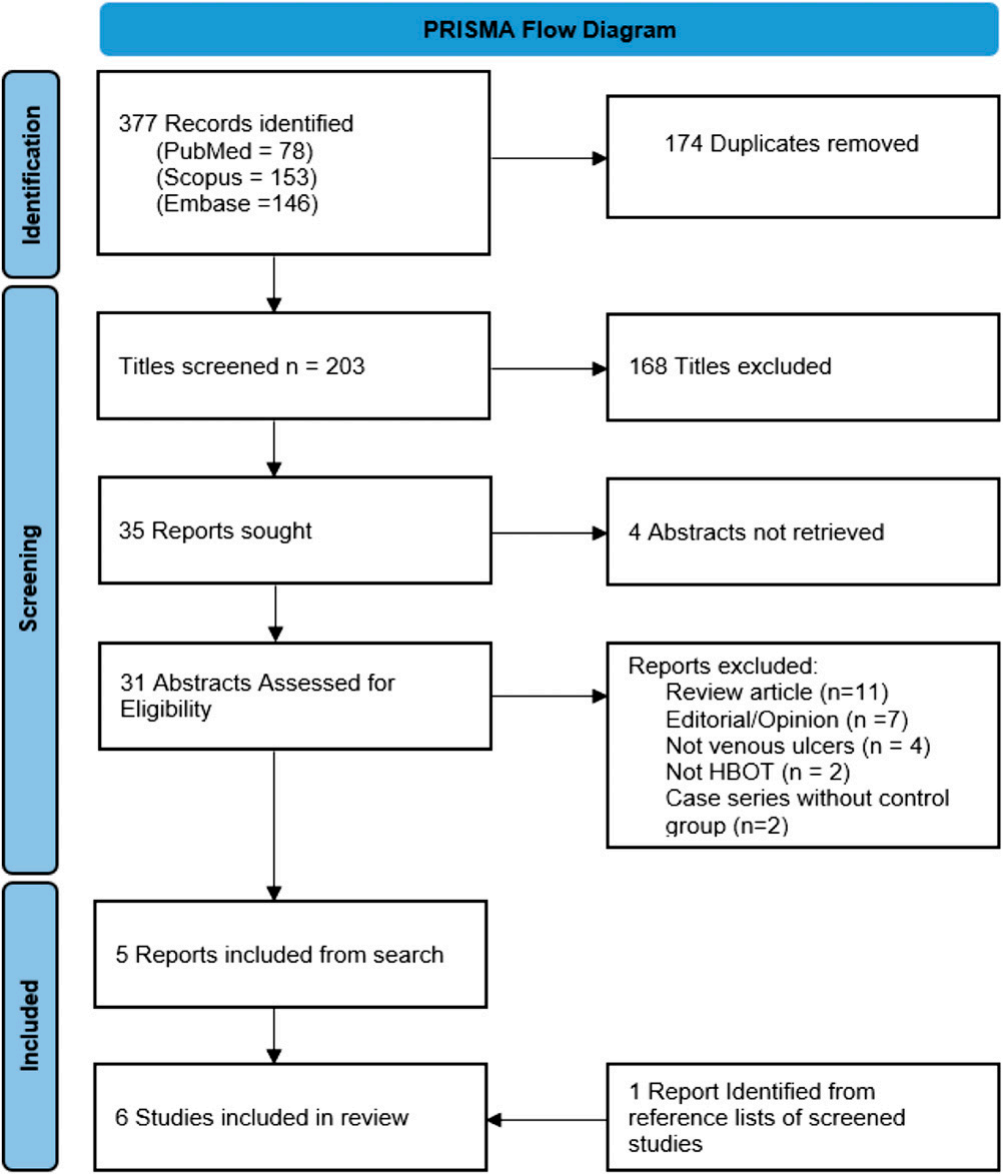
PRISMA flow diagram charting the literature search.

The database search of Scopus, PubMed, and Embase identified 377 titles for consideration. The last search was performed on July 7^th^, 2022. There were 203 unique publications remaining after removal of duplicates. 168 of these were excluded based on their title with a further 4 studies removed as no abstract could be obtained for review, 31 abstracts were screened for relevance. 5 studies were identified for inclusion.^11-15^Figure 1 illustrates a flow diagram of this process.

One of these included studies^15^ was found to be a published abstract which was never published as a full journal article. Efforts to contact the authors were unsuccessful. The decision was taken to include data from this abstract by Batora et al in the effect size meta-analysis, as sufficient primary outcome data was provided. This of course comes with the caveat that the risk of bias is higher due to the limited methodological information provided in the abstract. A further search through reference lists of included studies and excluded abstracts yielded 1 further relevant study, by Hammarlund et al.^16^

Two of the included studies contained a three-arm design. Ahmad et al^11^ included a cohort receiving laser-based treatment which was excluded from analysis. The control ‘standard wound care’ cohort and HBOT plus ‘standard wound care’ cohort were included. Longobardi et al^13^ primarily investigated markers of progress in VLU healing, but randomised participants to one non-HBOT cohort, and two cohorts of participants receiving different regimens of HBOT. Change in VLU area was reported, but it was reported as change in median VLU area. For the purposes of including this study in the meta-analysis these were converted to mean and standard deviation using the methods described by Luo et al^17^ and Wan et al.^18^

### Risk of Bias

**Figure 2.**
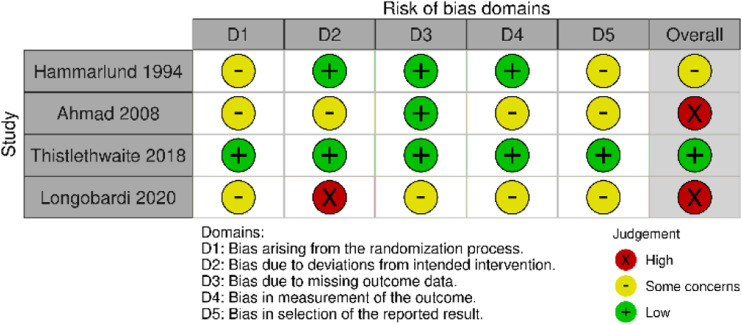
Risk of bias in randomized studies using RoB-2 tool.

**Figure 3.**
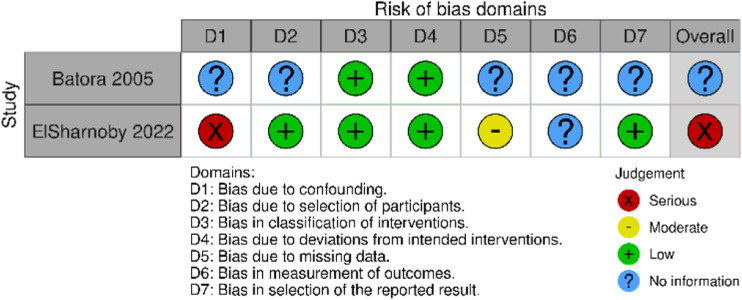
Risk of bias in non-randomized studies using the ROBINS-I tool.

Two separate tools were used to assess risk of bias. Figure 2 shows a graphic representation of the risk of bias in randomized studies included in the analysis, calculated using the RoB-2 tool and the risk of bias in non-randomized studies is shown in figure 3, calculated using ROBINS-I.

There were some concerns of bias in nearly all studies. Only the randomized trial by Thistlethwaite et al^12^ was found to have a low risk of bias. In general, concerns arose from the non-reporting of appropriate measures to prevent or limit bias, rather than an actual concern of bias in the study design. The one exception to this is ElSharnoby et al.^14^ This prospective study used patients’ ability to afford HBOT to determine segregation into treatment and control groups.

### Interventions

There is inconsistency in the schedule of HBOT treatment course administered across the included studies but with the exception of Ahmad et al, where the HBOT delivery is unclear, the treatments are broadly similar. The care given to control groups is much more variable however. Hammarlund et al and Thistlethwaite et al went as far as providing a sham treatment in the form of hyperbaric air, while Ahmad et al provided no compression or other therapy that would be considered standard therapy for VLU. The treatments received by both the HBOT and control groups in each study are summarized in Table 1.

**Table 1.** Summary of study characteristics, interventions for HBOT and control groups, follow up and reported rates of complete VLU healing.

Study Title	Study Type	Intervention	Control	Treatment Course	Follow up	No. healed/Sample	
						HBOT	Control
Hammarlund 1994	RCT	HBOT at 2.5 ATA for 90 minutes	Hyperbaric air at 2.5 ATA for 90 minutes	5 sessions/week over 6 weeks	6 weeks	0/8	0/8
Batora 2005	Comparative, allocation unspecified	HBOT at 1.8-2 (reported as .18-.2 MPa) for 72 minutes plus unspecified ‘conventional therapy’	Unspecified ‘conventional therapy’	‘Average 26 treatments’ over 6 weeks	6 weeks	0/13	0/12
Ahmad 2008	3-arm RCT	HBOT for 90 minutes daily	Wound cleansing twice daily	2 sessions/day for 5 weeks	5 weeks	0/10	0/10
Thistlethwaite 2018	RCT	HBOT at 2.4 ATA for 80 minutes, plus compression and decompression phases Compression therapy and dressings	Hyperbaric air at 1.05-1.2 ATA for 80 minutes. Compression therapy	30 sessions	12 weeks	6/14	6/15
Longobardi 2020	3-arm RCT	HBOT at 2.4 ATA for 66 minutes plus compression and decompression phases. Compression therapy	Compression therapy	One HBOT cohort received 2 sessions/day for 3 weeks, the other 1/day for 6 weeks	3 and 6 weeks	5/25	1/26
ElSharnoby 2022	Parallel prospective cohort study	HBOT at 2.5 ATA for 1 hour plus compression and decompression phases. Treatment of venous reflux. Compression therapy and dressings. Debridement where necessary	Treatment of venous reflux. Compression therapy and dressings. Debridement where necessary	5 sessions/week, 20-40 sessions	12 weeks	5/12	3/13

ATA = absolute Atmospheric pressure, mmHg = millimetres of Mercury.

### Outcome 1: Complete Healing of VLU

Only three of the studies reported any VLU having healed completely during the study period.^12-14^ Follow-up was heterogeneous, with some studies reporting a 12 week follow up period, while others reported outcomes only at the end of the treatment course of three, five, or six weeks. To minimise the effect of this, studies were pooled in two groups according to the follow-up period employed in each; a 5-6 week group and a 12 week group, with exclusion of one three week group. Table 1 shows the number of participants in each of the studies, and their outcomes. Pooled OR for this group was 5.39 (95%CI = .57 – 259.57) P = .1136, indicating no significant difference in VLU healing between the HBOT and non-HBOT cohorts.

**Figure 4.**
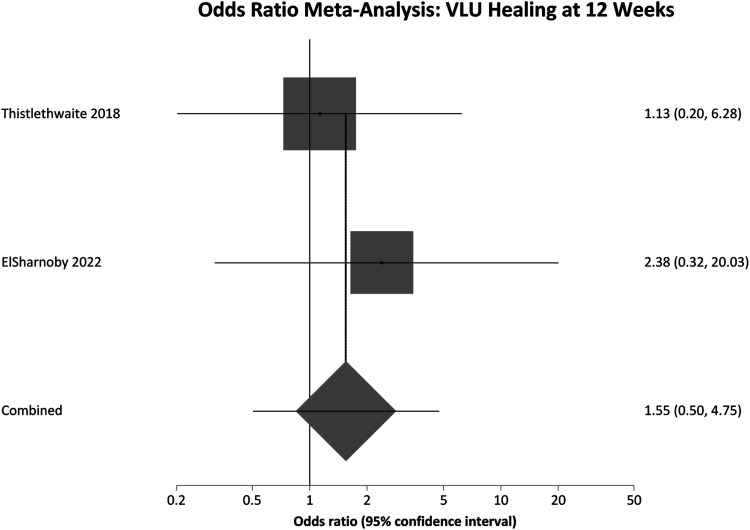
Odds ratio meta-analysis of complete VLU healing at 12 Weeks. OR >1 favours HBOT OR <1 favours control.

Thistlethwaite et al and ElSharnoby et al reported 12 weeks of follow up from the commencement of treatment. Pooled OR for this 12 week follow-up group was 1.54 (95% CI = .50 - 4.75) P = .4478, again showing no significant difference in VLU healing rates. Figure 4 contains a forest plot representing this pooled analysis.

Hammarlund et al and ElSharnoby et al both reported longer term follow up than that included in the meta-analysis. There were a number of losses to follow up in the study by Hammarlund et al from the end of treatment until delayed follow up at week 18 (16 weeks after commencement of treatment). While 2 VLU did heal completely within this period, any losses from such a small cohort were felt to pose a significant risk of bias. In order to avoid this, the initial end-of-treatment result was used in the primary analysis. ElSharnoby et al reported one year follow up and at one year, 10/12 VLU had healed in the HBOT arm, and 7/13 with venous intervention and compression.

### Outcome 2: Reduction in VLU Area

All included studies reported change in VLU area over time, but again pooled analysis is limited by variable follow up. Results were therefore separated again into two groups, interrogating the change in VLU area over 12 weeks of follow up (Table 2) and 5-6 weeks of follow up (Table 3). A further pooled analysis of change in VLU area over reported follow up was also performed (Table 4) to allow a single pooled analysis of all participants. Finally a pooled analysis of all RCTs was performed (Table 5)

**Table 2.** Reduction in VLU area in studies included in the effect size meta-analysis.

Study Title	Participants	HBOT		Control	
	(n = )	Mean Δ VLU Area	SD	Mean Δ VLU Area	SD	SMD		95%CI	
Thistlethwaite 2018	29	95(%)	6.53	54	6.78	5.98		4.28	7.69
ElSharnoby 2022	25	5.46	2.03	4.42	1.92	.51		−.29	1.31
Pooled	54					3.21		2.12	8.54

Δ VLU area = change in venous leg ulcer area SD = Standard deviation 95% CI = 95% confidence interval SMD = Standardized mean difference.

**Table 3.** Reduction in VLU area; effect size meta-analysis.

Study Title	Participants	HBOT		Control	
	(n = )	Mean Δ VLU Area	SD	Mean Δ VLU Area	SD	SMD	95%CI	
Hammarlund 1994	16	35.17(%)	17	2.7	11	2.18	.94	3.42
Batora 2005	25	60.8(%)	42.7	29.3	6.5	.98	.15	1.8
Ahmad 2008	20	1.68	.27	.77	.23	3.47	2.09	4.86
Longobardi 2020 (6 weeks)	48	67.46	40.05	59.72	33.86	.20	−.34	.76
Pooled	109					1.59	.30	2.89

Δ VLU area = change in venous leg ulcer area SD = Standard deviation 95% CI = 95% confidence interval SMD = Standardized mean difference.

**Table 4.** Reduction in VLU area in studies included in the effect size meta-analysis.

Study Title	Participants	HBOT		Control		SMD	95%CI	
	(n = )	Mean Δ VLU Area	SD	Mean Δ VLU Area	SD			
Hammarlund 1994	16	35.17(%)	17	2.7	11	2.18	.94	3.42
Batora 2005	25	60.8(%)	42.7	29.3	6.5	.98	.15	1.8
Ahmad 2008	20	1.68	.27	.77	.23	3.47	2.09	4.86
Thistlethwaite 2018	29	95(%)	6.53	54	6.78	5.98	4.28	7.69
Longobardi 2020 (6 weeks)	48	67.46(%)	40.05	59.72	33.86	.20	−.34	.76
Longobardi 2020 (3 weeks)	51	29.75(%)	26.48	31.3	15	−.06	−.63	.50
ElSharnoby 2022	25	5.46	2.03	4.42	1.92	.20	−.34	.76
Pooled	214					1.7	.60	2.79

Δ VLU area = change in venous leg ulcer area SD = Standard deviation 95% CI = 95% confidence interval SMD = Standardized mean difference.

**Table 5.** Effect Size meta-analysis of reduction in VLU area in Randomized Controlled Trials.

Study Title	Participants	HBOT		Control		SMD	95%CI	
	(n = )	Mean Δ VLU Area	SD	Mean Δ VLU Area	SD			
Hammarlund 1994	16	35.17(%)	17	2.7	11	2.18	.94	3.42
Ahmad 2008	20	1.68	.27	.77	.23	3.47	2.09	4.86
Thistlethwaite 2018	29	95(%)	6.53	54	6.78	5.98	4.28	7.69
Longobardi 2020 (6 weeks)	48	67.46	40.05	59.72	33.86	.20	−.34	.76
Longobardi 2020 (3 weeks)	51	29.75(%)	26.48	31.3	15	−.06	−.63	.50
Pooled	164					2.2	.55	3.84

Δ VLU area = change in venous leg ulcer area SD = Standard deviation 95% CI = 95% confidence interval SMD = Standardized mean difference.

.

**Figure 5.**
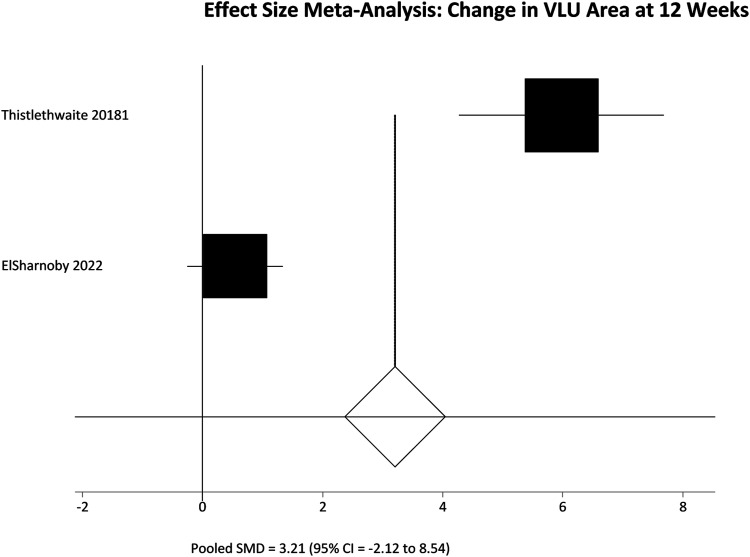
Effect size meta-analysis of change in ulcer area at 12 weeks. SMD >0 favours HBOT, SMD <0 favours control. In this case the pooled effect is not statistically significant.

**Figure 6.**
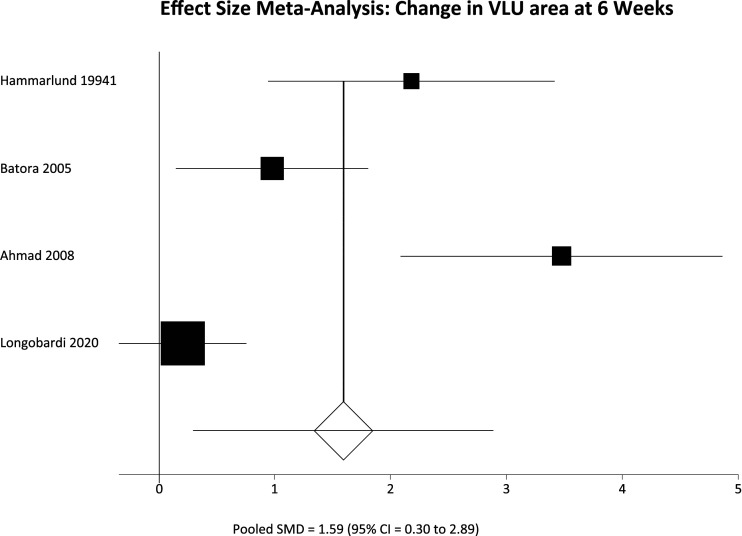
Effect size meta-analysis of change in ulcer area at 12 weeks. SMD >0 favours HBOT, SMD <0 favours control. In this case the pooled effect is statistically significant.

**Figure 7.**
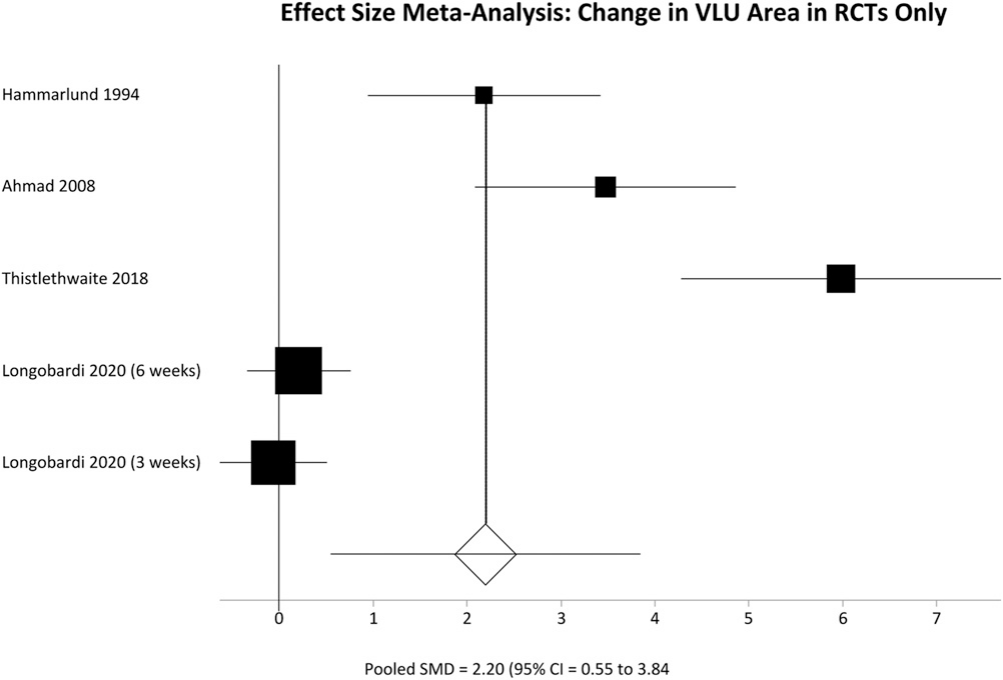
Effect size meta-analysis of change in ulcer area by the end of the study in included RCTs. SMD >0 favours HBOT, SMD <0 favours control. In this case the pooled effect is statistically significant.

Among participants with 12 weeks of follow up the pooled SMD was not significantly different; SMD = 3.21 (95% CI = −2.12 to 8.54) P = .2383. However, in the 5-6 week follow up group there was a significant difference in favour of the HBOT group, meaning greater reduction in VLU area; SMD = 1.59 (95% CI = .30 to 2.89) P = .016. These results are represented in figures 5 and 6 respectively. Finally pooling of only RCT data, again without segregation by follow up duration, also showed a statistically significant reduction in VLU area with HBOT; SMD = 2.20 (95% CI = .55 to 3.84) P = .0089. These results are represented in figure 7.

## Discussion

A quick glance at the included studies in this review might lead one to believe that that HBOT is a useful adjunct in the management of VLU. With the exception of Ahmad et al, the focus of whose trial was evaluation of laser therapy, all of the included studies concluded in favour of the use of HBOT, stating that HBOT ‘may have a role’, was ‘promising’, or was ‘a valuable adjunct’ in VLU healing. Our analysis however shows no statistically significant difference in the number of VLU healed by the addition of HBOT to existing wound care, suggesting limited, if any, clinical benefit. Our analysis is limited by the heterogeneity in multiple aspects of the included studies, in particular the outcome reporting measures and the duration of follow up.

Variable follow-up means direct comparisons are more difficult to make, in particular for a dichotomous outcome like complete healing of VLU. The lack of complete healing in many studies is probably at least partly attributable to brief follow up. This is borne out by the fact that included studies in which no VLU healed at all, involved no follow up beyond six weeks. However in the best-designed of the included studies, with adequate follow up and the lowest risk of bias, Thistlethwaite et al showed no difference in VLU healing rates at three months.

A serious flaw in an otherwise well-designed study by ElSharnoby et al is the use of patients’ ability to afford HBOT to dictate allocation. This has serious potential to confound any results because the ability of patients to afford expensive treatment suggests intuitively that this cohort must come from a higher socio-economic background than the control arm. Lower socioeconomic class has been shown to be significantly associated with reduced VLU healing.^19^ Even despite this potential confounding in favour of HBOT, the pooled analysis of healing rates at 12 weeks showed no significant difference between HBOT and controls.

Change in VLU area is a reasonable surrogate marker of treatment effect in VLU, which by their nature tend to be slow to fully heal. Change in VLU area outcomes were reported in some studies as an absolute change from baseline VLU area, while others reported the percentage change from baseline. This necessitated the use of SMD instead of weighted mean difference. While SMD is a perfectly valid statistical method for this comparison, it is much easier to assess the clinical relevance based on the weighted mean difference. The pooled analysis of these studies shows a statistically significant benefit to HBOT. It may well be the case that with sufficient follow up this statistical benefit would translate to a greater number of VLU healing completely, but given that this has not been seen in the groups with the longest follow up, the current data shows a statistically significant benefit without sufficient evidence of a clinically significant benefit.

There was a lack of homogenous control treatments across the included studies. This limits our ability to assess HBOT as an alternative to existing therapies. Only one study involved treatment of venous reflux which is increasingly becoming a standard part of management in VLU care, while only three studies involved compression therapy, which is well established as standard of care.

No indication was provided to suggest there was any stratification based on VLU size in any of the included studies. Given the small sample sizes this is probably unsurprising, but nevertheless has the potential to confound results.

A single, well designed, sufficiently powered RCT would provide much more compelling evidence than the aggregate of existing evidence. A power calculation based on the VLU healing rates at three months in our analysis suggests that such a trial would require close to a thousand participants.^20^ Given the costs associated with HBOT, such a trial seems unlikely.

### Limitations

The main limitations of this review are the small sample size in the included studies, the high risk of bias within most of the studies, the lack of consistency in control treatments and the short follow up periods. There are some concerns regarding the risk of bias for each study except for Thistlethwaite et al, while ElSharnoby et al is at particularly high risk because of their method of allocation, making socio-economic status a significant confounding factor. Finally, the inconsistency in control treatments limits the applicability of any findings in the real-world setting.

#### Interpretation

This review has found that while some existing evidence supports the use of HBOT as an adjunct in managing VLU, most of this evidence is of poor quality and therefore, questionable reliability. The addition of HBOT to existing treatment has not been shown to lead to any significant improvement in VLU healing rates in the short term. It has been shown that the addition of HBOT to existing treatment is associated with a statistically significant reduction in area of VLU vs controls, but this does not necessarily translate to a significant clinical benefit.

## Conclusion

Poor quality evidence from individual studies suggests a potential benefit from the use of HBOT in managing VLU, but the synthesis of this data does not support its widespread use.
